# Emerging *in vitro* models to study Merkel cell carcinoma pathobiology

**DOI:** 10.3389/fcell.2025.1691110

**Published:** 2025-10-10

**Authors:** Chiara Mazziotta, John Charles Rotondo

**Affiliations:** ^1^ Department of Medical Oncology, Dana-Farber Cancer Institute, Harvard Medical School, Boston, MA, United States; ^2^ IRCCS Ospedale Policlinico San Martino, Genova, Italy

**Keywords:** Merkel cell carcinoma, non-melanoma skin cancer, skin cancer, Merkel cell polyomavirus, oncogenic virus

## Abstract

Merkel cell carcinoma (MCC) is a rare but highly aggressive skin neoplasm, caused in approximately 80% of cases by the genomic integration of Merkel cell polyomavirus (MCPyV) and the expression of the viral small T antigen (sT) and large T antigen (LT) oncoproteins. Virus-negative tumors exhibit extensive UV-induced mutations. Despite such divergent molecular characteristics, the two etiologies share similar morphological and clinical features. The development of novel preclinical *in vitro* models that effectively recapitulate MCC pathobiology is essential for understanding the mechanisms of MCPyV infection and the cellular ancestry of MCC, a central topic of ongoing investigation and debate. This review provides a comprehensive overview of current two-dimensional (2D) and three-dimensional (3D) *in vitro* models developed to investigate the molecular and cellular mechanisms of MCC onset and progression. Continuous refinement of cell models that recapitulate MCC pathobiology is essential for advancing our understanding of the mechanisms of tumor onset and progression, thereby enhancing clinical applications for MCC patients.

## 1 Introduction

Merkel cell carcinoma (MCC) is a primary aggressive skin neuroendocrine tumor (Fakult et al., 2025). Despite being uncommon, its aggressive clinical course and limited therapies make MCC a clinical challenge. Approximately 80% of MCCs are associated with the DNA tumor virus Merkel cell polyomavirus (MCPyV) and considered MCPyV-positive (MCCP) (Feng et al., 2008). The remaining, virus-negative, MCC cases (MCCN) present extensive ultraviolet (UV) radiation-induced mutations (Harms et al., 2015; Wong et al., 2015). The partial understanding of the molecular characteristics of MCC, including viral oncogenesis and the currently unclear tumor origin cell (Mazziotta et al., 2025a), as well as the lack of robust diagnostic/prognostic biomarkers and therapeutic options collectively underscore the critical need for reliable preclinical models capable of recapitulating the MCC biology.

**FIGURE 1:**
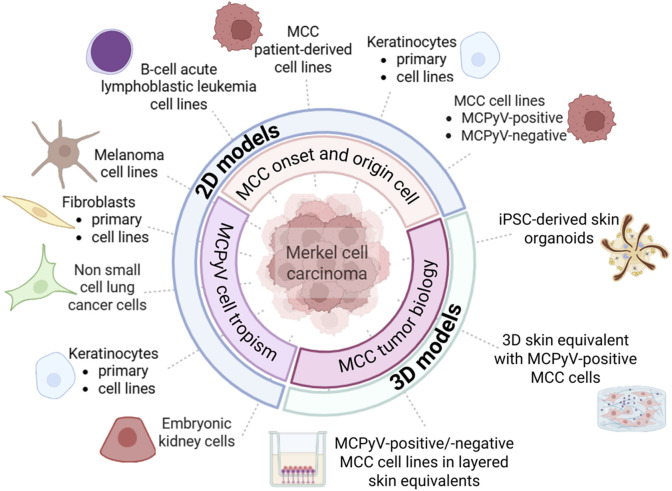
*In vitro* models designed for investigating the mechanisms of Merkel cell polyomavirus (MCPyV) infection and Merkel cell carcinoma (MCC) onset and progression. MCC-based two-dimensional (2D) cell models predominantly include (i) human fibroblasts, keratinocytes and embryonic kidney cells, (ii) MCPyV-positive and/or -negative MCC cell lines, as well as patient-derived MCC cell lines. Non-MCC tumor cell lines include melanoma, non-small cell lung carcinoma, and B-cell acute lymphoblastic leukemia cell lines. MCC-based three-dimensional (3D) cell models consist of co-culture systems/layered skin equivalents combining keratinocytes, fibroblasts, and MCC cell lines, as well as a human induced pluripotent stem cell (iPSC)–derived hair-bearing skin organoid system.

Cell-based *in vitro* systems are indispensable tools for studying cancer biology. Two-dimensional (2D) models have provided valuable insights into the MCPyV cell tropism, oncogene function, and molecular pathways sustaining MCC initiation and progression (Figure 1) (Loke et al., 2022). Studies using fibroblasts, keratinocytes, MCC cell lines and patient-derived cell lines have partially uncovered the mechanisms of MCPyV infection and viral oncoproteins functions. Moreover, only a few studies have established reliable MCC-based three-dimensional (3D) models (Figure 1). Although 3D models mimic the architectural and biological complexity of human skin, allowing the growth of MCC cells, they do not recapitulate the cellular heterogeneity and immune microenvironment of MCC.

In this review, we discuss the current *in vitro* approaches to study MCC pathobiology. We focus on both 2D and 3D cell systems, highlighting their contributions to understanding MCC, their limitations, and their future potential as tools to advance diagnostics, prognostics, and therapeutic strategies.

## 2 Merkel cell carcinoma

MCC is a rare neoplasm with an incidence of 0.3–1.6 cases/100,000 subjects/year globally (Fakult et al., 2025). Similar to other virus-driven tumors (Preti et al., 2020), MCCPs are characterized by the expression of viral oncoproteins LT/sT and LT truncation (tLT) which drive tumor onset/progression through p53/pRB inactivation (Mazziotta et al., 2025b). Inactivation of the same pathways, by UV-induced somatic mutations, is observed in MCCNs (Comut et al., 2025; Tribble et al., 2025). MCCNs exhibit a high mutation burden (Goh et al., 2016; Tribble et al., 2025). Epigenetic alterations have been reported (Mazziotta et al., 2023b). MCC risk factors include chronic UV-radiation exposure and conditions of immune impairment and/or immunosuppression.

MCC typically manifests as firm, flesh-colored cutaneus or subcutaneus nodules and does not show pre-tumor lesions (Scotti et al., 2025). Routine staging with sentinel lymph node biopsy (SLNB) is advised for stage I-II patients (Gauci et al., 2022). The immunohistochemical profile of MCC includes neuroendocrine markers (chromogranin, synaptophysin, NCAM1/CD56) and epithelial markers (cytokeratin AE1/AE3, CAM5.2, CK20). In contrast, MCC is usually negative for markers observed in other tumors, such as TTF-1, carcinoembryonic antigens, keratin 7, S-100 proteins, PMEL, and lymphoma-specific lymphocyte markers. Prognosis is influenced by extracutaneous tumor extension and regional or distant spread, with lymph node status as a key independent indicator. Markers with prognostic value include infiltrating immune cells (Becker et al., 2024), and KIT, BIRC5, hedgehog proteins and p53/p63 expressions. MCCP patients present a more favorable prognosis compared to virus-negative patients (Liu et al., 2025).

MCC presents a 5-year survival rate of 63% (Gauci et al., 2022). The standard approach for localized/regional tumors is surgical excision followed by adjuvant radiotherapy (Stachyra et al., 2021). Radiotherapy is recommended for patients with nodal involvement, while adjuvant chemotherapy is uncommon. Guidelines for systemic therapy of advanced/metastatic MCC are drawn from data derived from other neuroendocrine tumors, such as small-cell lung carcinoma (Gauci et al., 2022). Immunotherapies with programmed death-1/ligand-1 inhibitors (anti-PD-(L)1) such as avelumab (anti-PD-L1), pembrolizumab, and nivolumab (both anti-PD-1) have shown promising efficacy for advanced MCC (Paulson and Bhatia, 2018; Kakish et al., 2024; Mo et al., 2025). However, about half of patients experience primary/secondary resistance (Fojnica et al., 2023). The establishment of novel *in vitro* MCC models holds significant potential for advancing therapeutic options.

## 3 Two-dimensional models

### 3.1 Models for studying MCPyV cell tropism and viral life cycle

To date, infectious MCPyV particles have not been isolated from naturally infected human cells. The clonal integration of MCPyV DNA, which precludes productive replication, suggests that viral tropism may be restricted to a non-MCC cell type. Early experiments with recombinant MCPyV plasmids demonstrated the ability of MCPyV to infect fibroblast-like PFSK-I cells derived from a neuroectodermal tumor, H1299 non-small cell lung carcinoma (NSCLC) cells, and HEK293 human embryonic kidney cells (Neumann et al., 2011). These findings suggest that different human cell types could support MCPyV infection. To identify the cell types that support MCPyV entry, an early study screened numerous non-tumor human cells and 60 human cancer cell lines from the NCI-60 panel, finding that only melanoma cells and primary keratinocytes permitted viral entry (Schowalter et al., 2012). More recently, Liu et al. demonstrated MCPyV entry in keratinocytes and fibroblasts, with complete replication occurring exclusively in fibroblasts (Liu et al., 2016). In particular, the study describes a dermal fibroblast cell culture model in which MCPyV entry and replication were shown to be facilitated by the matrix metalloproteinase-mediated activation of the WNT/β-catenin pathway. Moreover, treatment with the MEK inhibitor trametinib suppressed viral transcription and replication, highlighting a potential therapeutic strategy based on targeting MCPyV. These findings demonstrate that human dermal fibroblasts support MCPyV entry, gene expression, and replication (Liu et al., 2016).

Additional findings indicate that human foreskin fibroblasts and marrow- or adipose-derived mesenchymal stem cells can support MCPyV infection (Abere et al., 2022). Subsequent studies revealed that viral DNA replication in fibroblasts can induce genomic stress, cell cycle arrest, and senescence (Siebels et al., 2020), raising doubts about reliability of earlier observations. More recent evidence, however, has established fibroblasts as a robust model for investigating MCPyV and MCC, confirming their ability to support the complete viral infectious cycle and sustain early gene expression. For instance, a recent functional study employing human dermal fibroblasts (HDFs) reported that MCPyV LT/sT transcription is regulated by the histone acetyltransferases p300 and CBP, which coactivate the transcription factor NF-κB to promote viral gene expression (Yang et al., 2023). Another recent study using human foreskin fibroblasts explored the mechanisms of action of various sT from multiple PyVs, including MCPyV. The study demonstrated that only MCPyV sT accomplishes cellular transformation by localizing to the nucleus (Thevenin et al., 2024).

Additional NSCLC cell lines, particularly A549, demonstrated susceptibility to MCPyV infection and permissivity to viral replication, making them a frequent model for studying viral entry (Schowalter et al., 2011; Becker et al., 2019; Wang et al., 2025). A recent study using A549 cells demonstrated that unlike other polyomaviruses, MCPyV does not require the nuclear pore complex during entry, while instead taking advantage cell cycle–linked nuclear envelope breakdown, a process driven by the VP1 capsid protein, to access the nucleus (Wang et al., 2025).

Concerning embryonic kidney cells, a recent study with HEK293 cells and focused on evaluating the relationship between MCPyV oncoproteins and a component of the Wnt/β-catenin signaling pathway, namely CK1α, demonstrated that HEK293 can be transfected with MCPyV LT. This study underlines not only that kinase pathways are indispensable for governing MCPyV infection and life cycle but also supports the use of embryonic kidney cells for studying MCPyV (Pham and Kwun, 2024).

These studies investigated the potential of MCPyV to infect multiple human cell types, with fibroblasts identified as the sole cell type supporting the complete viral life cycle. These cells currently represent a reliable cell model for investigating MCPyV and/or MCC. However, recent evidence suggests that the host cell supporting MCPyV replication and the MCC originating cell may actually be distinct cell types (Landazuri Vinueza et al., 2025).

### 3.2 Models for studying the MCC cellular origin and oncogenesis

Identifying the cell of origin for MCC is an active area of investigation and debate (Jeremian et al., 2025; Mazziotta et al., 2025a). Merkel cells, skin mechanoreceptors (Hartschuh et al., 1984; Hartschuh et al., 1989; Cervellera et al., 2025), have traditionally been considered the MCC origin cells. The co-expression of neuroendocrine, epithelial, and B-lymphoid markers in MCC cells, together with the abovementioned permissiveness of fibroblasts to MCPyV infection, supports the idea of a multilinear origin and disputes the traditional Merkel cell–derived model. It has been proposed that MCCP cells may originate from dermal fibroblasts, undifferentiated Merkel cell precursors and pro/pre- or pre-B lymphocytes. Multiple comparative molecular analyses between MCCP and MCCN cells explored these aspects (Kumar et al., 2018; Gravemeyer et al., 2021; Harms et al., 2022; Mazziotta et al., 2023a; Mazziotta et al., 2023b; Passerini et al., 2025). Functional approaches for investigating MCCP focus on reversing malignancy by silencing MCPyV oncogenes, or on inducing transformation of non-MCC cells via viral oncogene expression. For instance, MCPyV LT knockdown has been successfully achieved in multiple MCCP cell lines, including MKL-1, MS-1 and CVG-1, co-cultured with keratinocytes (Harold et al., 2019). In particular, upon MCPyV LT knockdown, MCCP cells transitioned toward a neuronal-like differentiated phenotype, a process dependent on the presence of keratinocytes. These cells expressed canonical Merkel cell markers ATOH1, SOX2, HES6, and KRT20 and developed neurite-like extensions, suggesting that altered Merkel cell differentiation pathways may be associated with MCCP initiation. An additional model with lentiviruses encoding MCPyV oncoproteins has been employed to transduce primary human keratinocytes (Kervarrec et al., 2020). Keratinocytes co-expressing MCPyV oncoproteins and the developmental factor GLI1, displayed positivity for Merkel cell markers SOX2, K8, and K20 and advanced Merkel cell phenotypes including a floating morphology, similarly to that of MCCP cells. An *in vivo* model of SOX9-expressing epidermal cells (SOX9^+^) as Merkel cell progenitors has been employed for evaluating whether these cells can be reprogrammed toward the neuroendocrine lineage by MCPyV oncoproteins, as occurring in MCC (Weber et al., 2023). MCPyV LT/sT expression drove SOX9^+^ cells to an enforced neuroendocrine and Merkel cell lineage reprogramming. Consistently, LT-negative MCC tumors tend to co-express genes related to squamous differentiation, especially SOX9, and the cell cycle such as MYC and CDK6 (Torre-Castro et al., 2024). These *in vitro* models collectively support the hypothesis that SOX9^+^ Merkel cell progenitors might originate MCC.

A transcriptome-based classification of MCC tumors, regardless of MCPyV status, suggests that the silencing of Hippo pathway regulators YAP1 and WWTR1 is crucial for MCCP development (Frost et al., 2023). The study, conducted with MCC cell lines and patient-derived cell lines, reported that YAP1 and WWTR1 expression in MCC tumors is inversely correlated with neuroendocrine markers. Functional data from MKL-1 cells showed that YAP1/WWTR1 silencing is essential for MCCP development through LT regulation. The study highlights the novel concept of exclusivity between YAP1/WWTR1 and neuroendocrine transcriptional programs in MCC cells.

Growing research has explored the multifaceted tumor-promoting activities of MCPyV oncoproteins utilizing MCC cell lines, MCC patient-derived cell lines and non-MCC tumor cells. A recent study with MCC cell lines demonstrated that LT/sT can target SET/PP2A complex to promote cellular proliferation/migration (Gupta et al., 2024). Investigations into DNA damage stress responses in MCC cells highlighted the role of tLT and the lysine methyltransferase EHMT2 in maintaining tumor cell genomic integrity (Bachiri et al., 2025). A model of MCC patient-derived cell lines revealed that LT can suppress autophagy via Kit signaling, a crucial survival mechanism for tumor cells (Shi et al., 2025). Intriguingly, a recent study employed a B-cell acute lymphoblastic leukemia (ALL) cell line namely REH, along with the MCCN cell line MCC13, for evaluating the role of MCPyV in driving MCC development. Transfection experiments indicate that LT can drive DNA methylation and gene expression changes in both cell lines, while sT exerts effects only in REH cells (Macamo et al., 2024). Collectively, these MCC cell models underscored the multifaceted activities of MCPyV oncoproteins.

Several models have been established to explore the functional relevance of miRNAs in MCC, with particular attention to hsa-miR-375, hsa-miR-200c-141, hsa-miR-183-96-182, hsa-miR-203, hsa-miR-30a-3p, hsa-miR-30a-5p, and hsa-miR-20a-5p (Xie et al., 2014; Abraham et al., 2016; Kumar et al., 2018; Kumar et al., 2020; Orouji et al., 2020; Durante et al., 2021; Fan et al., 2021; Gravemeyer et al., 2021; Durante et al., 2022; Mazziotta et al., 2023b). These approaches largely rely on MCCP and MCCN cell models subjected to forced miRNA overexpression and/or knockdown, allowing investigation of downstream targets, signaling pathways, and resulting phenotypic changes.

## 4 Three-dimensional models

Only a limited number of MCC-based 3D models have been developed. Loke et al. developed a co-culture comprising keratinocytes, fibroblasts, and MKL-1 cells (Loke et al., 2021), starting from a previously established 3D skin system for Human papillomavirus (Lee et al., 2016). MCCP-like lesions have been generated either by positioning them as a transition layer between dermal and epithelial compartments or generated by embedding MKL-1 cells within the dermal equivalent. Tumor cells expressed canonical MCC markers and exhibited proliferative capacity.

Subsequent work expanded these approaches by incorporating variable combinations of keratinocytes, fibroblasts, and tumor cells, thereby offering a more comprehensive model of MCC biology (Temblador et al., 2022). MCCP cell lines MKL-1, MS-1 and WaGa and MCCN cell lines MCC14/2, MCC13 and MCC26 have been either cultured atop fibroblast-derived dermal equivalents or pre-mixed with keratinocytes and/or fibroblasts before seeding. While MCCN cells proliferated and retained MCC marker expression, co-culture with keratinocytes facilitated the development of MCCP/MCCN-like lesions within the epithelial layer. Lesions resembling MCCN, but not MCCP, were instead observed in the dermal equivalent, closely mimicking *in vivo* MCC phenotypes, unlike earlier models (Loke et al., 2021).

The development of complex 3D tissue models derived from human induced pluripotent stem cells (iPSCs), including skin organoids, may represent a promising approach to investigate viral pathogenesis dynamics. An iPSC-derived hair-bearing skin organoid (SkO) system has been recently established for investigating MCPyV infection, progression and spread (Albertini et al., 2025). Using bulk-, single cell and spatial-transcriptomics, combined with immunostaining and nucleic acid hybridization technologies, the system demonstrated that MCPyV can persist in a quasi-latent state within the majority of dermal fibroblasts carrying the viral genome. The authors subsequently identified papillary fibroblasts and dermal sheath fibroblasts as capable of viral replication and progeny production. iPSC-derived SkOs demonstrated the potential to support infection and long-term persistence of the virus similarly to physiological conditions in humans. The iPSC-derived SkO model holds significant potential in improving the development of intervention strategies for both chronic MCPyV infection and pathogenesis. Although still limited in number, these 3D models have the potential to bridge the gap between simplified 2D systems and the complex *in vivo* MCC microenvironment, highlighting their ability to accurately study MCPyV and recapitulate MCC pathobiology.

## 5 Conclusion and future Perspectives

The development of reliable *in vitro* models has been crucial for advancing our understanding of MCC biology (Pedersen et al., 2024). Substantial progress has been made in establishing 2D models, yet significant challenges persist. The primary challenges in establishing reliable MCC cell models include identifying the specific MCPyV target cell, determining the post-infection cell fate, and clarifying the MCC cell of origin. Further 2D models with other human cell types, such as pre-/pro-B cells, fibroblasts or dermal progenitors should be developed to test current hypotheses. Given the potential of 3D models in recapitulating skin biology, they are ideal for studying the complete MCPyV life cycle and transformative potential. Fibroblasts, keratinocytes and MCC cells co-cultures have successfully generated MCC-like lesions. The integration of immune cells into organoid platforms could further enhance their potential to model tumor–immune interactions, a critical aspect since immunotherapies are gaining importance in MCC. The already established 2D MCC cell systems including genetically manipulated MCC cells and/or early epidermal stem cells and unipotent Merkel cell progenitors should be further employed for investigating the transformative process throughout 3D models. To better recapitulate the architectural complexity, cell heterogeneity and immune microenvironment of MCC, the establishment of novel 3D systems such as patient-derived organoids, 3D bioprinted skin constructs harboring MCC tumors, and tumor-on-a-chip microfluidic platforms, as already established for other tumors (Jubelin et al., 2022), should be considered.

Numerous studies have investigated the activity of antitumor compounds across multiple preclinical models, employing both established MCC cell lines (Mazziotta et al., 2024; Narayanan et al., 2024), and patient-derived cell lines (Ananthapadmanabhan et al., 2022; Kunika et al., 2025). These models are widely used across a variety of cancer types (Stomper et al., 2021; Vincenzi et al., 2021; Pelos et al., 2024), and provide robust platforms for potentially advancing investigations with subsequent *in vivo* models. MCC 3D models and patient-derived organoids could also be used to evaluate the efficacy of antitumor agents.

Significant limitations remain. The rarity of MCC limits access to primary tumor material, hindering the standardized establishment of stable cell systems. Both 2D and 3D models may therefore be limited in power and generalizability until larger, multi-center studies are performed (Forsythe et al., 2022). Most MCC models focus on the more prevalent MCCP tumors, while developing MCCN models is essential to elucidate mechanisms of this rarer subtype. Moreover, the few available cell lines do not capture the inter-patient heterogeneity of MCC, restricting *in vitro* modeling of molecular subtypes, histopathological features, and clinical behaviors. Using multiple MCC cell lines, potentially sourced through multicenter cell line biobanks, is therefore recommended to enhance the biological relevance and experimental reproducibility. Several factors underscore the challenges in developing MCC cell models that recapitulate tumor heterogeneity and microenvironmental interactions. 2D cancer cell cultures lack morphology, heterogeneous phenotypes, immune component and extracellular matrix, which are characteristics of 3D systems. Organoid models better mimic tumor architecture and cell–cell interactions but face challenges of reproducibility, handling, standardized analyses. Moreover, both 2D and 3D models generally lack immune and stromal elements, restricting translational value for mechanistic studies and drug testing. These models need to be further refined and optimized to better recapitulate the MCC immune microenvironment.

In conclusion, the continuous development and optimization of *in vitro* models remain essential for advancing knowledge of MCC biology. These models will be instrumental in bridging the gap between molecular discoveries and clinical translation, ultimately guiding the development of improved diagnostic tools and therapeutic strategies for MCC patients. Further progress in the development of cell-based models would improve the understanding of MCC pathobiology.
